# The Foster-Brothers

**Published:** 1881-04

**Authors:** 


### THE FOSTER-BROTHERS.

HE SAT facing the river and the west, where the silver crescent hung above the Missouri hills. Forced by her silence to say something, he said hastily. "Turn to the left and look at the new moon. It will bring you great good luck this month."

He seemed to himself to speak involuntarily—he listened with interest to his own words.

She turned to him. "I will not look at the moon. I want no luck. I am happy enough. Besides," she added, with a smile as delicate as the starlight, "I can see the moon now, in your eyes."

"Can you see anything else there!"

She turned away, her heart beating with a vague apprehension.

"Can you see that I love you? that I worship you? that my free will is gone? that—I love you?"

She covered her face with her hands.

"I have been living here in a dream. I see now what it means. It is fatal for good or ill. My whole life fails if I go from here without you. Marie, will you go with me?"

He paused for a moment.

"If you say nothing I shall go mad with a false hope."

She turned her glowing face toward him. Even in that dim light the radiance of a new and wonderful happiness shone in her perfect features with a faint opaline gleam. But her manner was more quiet and self-possessed than usual as she said:

"I wish you would bring my father to me."

"But, Miss Des Ponts, shall I not have one word—?"

"Please bring my father," she insisted, in a low, appealing tone that there was no resisting.

When they came out she went to her father and leaned upon his arm. In spite of his intense anxiety Brydges could not but admire the statuesque beauty of the group. So Iphigenia must have clung at Aulis to the King of Men; and so the greatest of the Greeks must have folded in his strong arm the most beautiful, protecting her against all the world but him.

"Victor, Mr. Brydges has asked me to be his wife. I will not answer without your sanction."

"My darling, follow your heart, and you will make me only less happy than yourself."

She gave Clarence her hand and said, "I never dreamed I could love two beings as I love my father and you."

Des Ponts went in, with a strange contest of pleasure and pain in his heart, and left the lovers in the dim light of the setting moon.

Before Clarence slept he wrote a long letter to his father, announcing his engagement and giving many details of the character and position of the Des Ponts. He did not ask his father's consent formally. He was so thoroughly convinced of the propriety of his action that he would have disregarded his father's absolute veto. But, nevertheless, he awaited with some interest Mr. Brydges' reply.

Among the occasional visitors at Fort Johnstone was a friend of Mr. Marshall, a lawyer of Moscow, with whose graceful, though somewhat formal bearing, measured speech, and thorough moderation, as well in speech as in opinion, Clarence was much impressed. One night when this gentleman was gone Mrs. Marshall said, "You would scarcely suppose that Mr. X—— had been tried for murder and acquitted by a quibble?"

Brydges expressed his surprise.

"He was one of the slayers of the Mormon prophet Joe Smith, at our county jail."

"By-the-way, Clarence," said Cade, "would you not like to drive out to-morrow and see where the church seed was spilled?"

Brydges gladly assented; and the young men drove over the prairie in the cool of the morning. After visiting the scene of the tragedy, Clarence, who had been remarkably taciturn and thoughtful all the morning, said abruptly:

"I wish you would present me to your County Clerk. I want a marriage license."

"Bravo!" shouted Cade. "I see you never want to come here again. But it is not necessary. I can get your license whenever you want it."

"I may want it very soon. You know, Cade, I am fixed upon this marriage. No opposition from my father could change me. But Des Ponts and Marie are very spirited people. If my father should be whimsical enough to object, they might take umbrage. But if I could be married at once and go home with Marie, Monsieur mon père would yield to her beauty and grace as readily as did Monsieur his son."

"You are Sam Slick and Machiavel rolled into one. Here we are at the court-house."

They got the license and drove home.

"Do you think they will consent to this chain-lightning plan of yours?

"I do not think Marie will require much time. I imagine that in Thebes she has been uncorrupted by the mania of shopping. I *am* a little afraid of Des Ponts."

But to his surprise Des Ponts assented with alacrity to an immediate marriage. He said he should soon be compelled to make a journey to the East—perhaps to Europe. He would be glad to see his daughter's happiness secured before he started.

Marie at first protested loudly, but finding no sympathy in her lover or her father she flew to Mrs. Marshall, but found her equally hard-hearted.

"Nonsense, child," said the merry old lady. "We can make you lovely in half an hour. Why, Marshall proposed to me in this very fort while the long-roll was beating one evening, and we were married before the guard was out."

Marie yielded with a pretty girlish grace, that had come in these last days as the finishing charm of a character formerly, perhaps, too firm and self-reliant. Des Ponts' restlessness and anxiety seemed to increase hour by hour. He spent much of his time in Thebes arranging his papers and closing up pending affairs. The flood had now subsided, and a half-drowned slimy life began to move sluggishly through the soft black streets. He alleged to every one urgent business as an excuse for his sudden and unwonted activity. He did not say distinctly where he was going, but spoke sometimes of New Orleans and oftener of Europe.

"Why will you not go with us?" asked Clarence.

"Later I hope to join you," he would answer, with a smile heart-breaking in the sadness that tried to be gay.

The trunks were packed and sent to the wharf. The bride stood on the verandah dressed for travel, as bright in her blushes and tears as a morning of April. Good Mrs. Marshall, quite melted by her sympathetic happiness, was laughing and crying together, and giving Marie a world of motherly last words.

A red-faced, sleepy-eyed youth came up and asked for Mr. Brydges. "Here's a tallygraft fur him."

Clarence opened the envelope hastily and said, "How unfortunate! Father has started to come to the wedding, and is at St. Louis—says he will leave on this evening's packet. I must send him a telegram to wait there for us."

He wrote the dispatch, and was about handing it to the shabby messenger when Colonel Marshall said, "I will send it to the office by Thomas. I don't think this beery youth is quite awake yet."

"I regret, Mr. Des Ponts," said Clarence, "that you are not to meet my father here. You may be acquaintances, after all. My father lived in Savannah some thirty years ago."

At this moment the steamer rounded the point to the north, and her shrill whistle broke off the conversation. Des Ponts turned ashy pale. His daughter clung to him one instant. There was a confusion of hurried farewells, and the young people drove away to the wharf. In the slanting sunshine of the early morning the clouds of dust raised by their carriage-wheels turned to a rosy halo in which they passed out of sight.

There was a moment of silence. It was broken by Des Ponts, who said, in a husky voice, "Good-bye, my old friends. I shall not attempt to thank you enough for your life-long good-dess to me and mine."

He took a packet from his paletot and handed it to Colonel Marshall. "This contains my will and one or two other trifles. I am going away for awhile—"

"But not immediately?"

"Yes. I can finish to-day the little matters that remain in Thebes. I want to—" He paused, as if in doubt; then continued, in a manner strangely different from his usual one, "I have been tormented all night by impish dreams; and this morning I feel all abroad. I was always rather a lazy man, but the prospect of an absolute *far niente* is by no means alluring. My work in life is done—" Seeing the look of distress on the face of his friends, he forced a smile and said, "Perhaps I shall learn to enjoy the play-time."

"Yes, we will talk that over at tea," said Mrs. Marshall. "You must certainly stay with us until your departure."

"Well, well, I will come back to-night."

Then, as he was going to the door, he turned and said, "Colonel, it has been a quarter of a century since I heard Booth, yet all this morning I have been haunted by his tones in the words: 'Thou wouldst not think how ill all's here about my heart.' And the other phrase: 'If it be now, 'tis not to come; if it be not to come, it will be now; *if it be not now, yet it will come.*'"

That afternoon Cade Marshall picked up in the library the telegram which Brydges had dropped, and exclaimed:

"Here's a contretemps! This telegram arrived yesterday. That tipsy rascal forgot to deliver it."

"That *is* awkward," said the Colonel. "We can do nothing now but go to the landing for Mr. Brydges and explain the mistake. It is not so bad, after all. He can meet Des Ponts here, and will be sure to like him. Marie and Clarence can pass a day or two pleasantly in St. Louis."

Des Ponts did not return to tea. The steamer was late in coming. All the afternoon the Marshalls watched for it. It was already dark when the Colonel saw the red head-light shining far down the river. Even while he looked he saw, to his horror, a bright tongue of flame dart from the lower deck and rapidly climb up the side. It seemed but a moment until the steamer was wrapped in a lurid blaze that blotted out the moonlight and gleamed balefully over the low bottoms to the distant bluffs.

Des Ponts saw it also, midway of the river. He had been detained in Thebes until he was too late for the ferry, and had taken his skiff to row across in the cool fresh night. The fine air and the exercise of rowing kindled his blood, and he threw off the depression that had weighed upon him during the day. Resting on his oars a moment, and looking about him, he saw the steamer coming around the bar, and in an instant observed the red jet of fire that spurted from the engine-deck. He turned and rowed to the spot as fast as his strong arms and the swift current could carry him, but before he was there the doomed vessel, all pine and paint and tinder, a most delicate morsel for the fire-fiend, was enveloped in a shroud of flame. The water was filled with struggling and drowning passengers. One of these Des Ponts seized, and, in doing so, dropped an oar. By the time he had lifted the exhausted man into the skiff they had drifted some distance astern of the blazing wreck. Perceiving he had lost an oar, he stood in the stern of his boat to scull back to where others were still struggling. The glare of the conflagration was full in his face. His hat had fallen, and his fine head was brilliantly relieved against the dark like a portrait of Rembrandt.

The man he had saved, lying in the bow, burst out into a loud laugh.

Des Ponts felt the blood freezing in his veins. A shudder passed over his frame so powerful that the boat trembled with it. The horror that had dogged him through life was there, open-mouthed, in his path. But he still held his oar with a grip of iron, and stood as motionless as marble.

"Sam, your'e a d—d lucky nigger! You have saved your hide forty by picking me up to-night."

Des Ponts trembled again, with the ghost of the old slavish terror that stirred in his soul. He remembered, with horrible humiliation, the story of Herodotus, where the Scythians quelled a triumphant army of slaves by dropping their swords and attacking with their riding-whips. He felt in every fibre of his being that the story was true.

"Come, come, Sam, don't be sulky," said the man in the bow, with a tone meant to be good-natured. "Row me ashore and leave those rats to swim for it. I sha'n't be hard on you if you behave yourself. How is Clarence?"

"You son has married my daughter," said Des Ponts.

"Capital!" laughed Brydges. "The girl will come along without any row."

These brutal words recalled Des Ponts to himself. A light which was almost triumph came into his eyes.

"Victor Brydges," he said, firmly and quietly, "I do not wish to play at comedy with you. I am your boy Sam. I have no legal existence, you say; I have no child, no name. Granting this, your son is legally married to the natural daughter of Miss Julia Shelby, of Glenarthur."

Brydges saw that this calm statement was incontrovertible. In his sullen rage he fumbled in his waistband, and drawing a dirk, sprang at Des Ponts and struck him in the face. Des Ponts seized and disarmed him, throwing him back heavily into the bow. He tossed the dirk, wet with his own blood, into the river.

"You see how strong I am, and how weak you are, Victor Brydges. If you come at me again I will kill you."

In the slight scuffle he had regained his self-possession. He seated himself on one of the thwarts, facing Brydges. The boat floated with the current. They were nearly out of the glare of the burning boat, but there was light enough to show the firm-set face of Des Ponts, where the streaming blood drew lines of crimson across its ghastly pallor. There were no fear in it now, only the paleness of cool and self-conscious desperation.

"In fifteen minutes," he said, "this current will carry us ashore on Fox Island. We must settle our differences in that time. Unless you come to my terms you shall never leave this boat alive."

"Let's hear your terms," said Brydges.

"Our children are married and happy. Let them alone. I have given my daughter a dowry of one hundred thousand dollars. I will give you the highest market-price for myself. In return, you will bind yourself by oath, in writing, to silence—that is all."

"Sam! I will be candid with you. I think you are lying. You have not so much money. If you have, it is mine already. Consent to break up this marriage, pay me for yourself and the girl, and we will fix it up without scandal. I don't forget we are foster-brothers. You have received much kindness from my family—"

"Stop there! I was brought up with you to serve you. I learned to read, helping you. I learned French to be useful to you when you went abroad. I studied at the University to coach you through. I got my degree, and you failed. When at last I offered to buy myself and remain in France, you cursed me and struck me; and after that you were fool enough to trust me. You made me use my credit at the Prefecture of Police to get you a passport under the name of Des Ponts, to assist you in some scandalous intrigue. I swear that until I had that passport in my hands I never dreamed of running away.

"It has played the devil with you at last. I should never have found you but for that name in Clarence's letter."

"I met and married my wife under that name. I had made it honorable, and could not change it."

"Well, what do you say to my terms?"

"Victor Brydges," said Des Ponts, with solemn earnestness, "our children are married and happy. I am ready to die for the good of my daughter, and you must die for the good of your son, unless you accept my offer."

While he spoke Brydges was looking about him. He saw the boat had drifted so near to the low, willowy island that he could easily swim that distance. He felt himself unequal to another grapple with Des Ponts. His whole soul revolted against making any compromise with his slave. He slid over the boat's side with the quickness of an otter. Des Ponts started to his feet. "God have mercy on us both!" he murmured, hoarsely. As the head of Brydges came to the surface he plunged into the rapid current, and the foster-brothers went to the bottom locked in each other's arms.

They were found in the same posture two days later, lying on the shining sand. The Marshalls mourned and buried them side by side. Mrs. Marshall concluded a long and tender letter to Marie with these words:

"My dear children, from their graves your beloved fathers—

one of whom lost his precious life in his noble effort to rescue the other from the waves—exhort you continually to love one another."

### FOR THE CURIOUS.

Mezzotinto owed its invention to the simple accident of the gun barrel of a sentry becoming rusted with dew.

The process of whitening sugar was discovered in a curious way. A hen that had gone through a clay puddle went with her muddy feet into a sugar-house. She left her tracks on a pile of sugar. It was noticed that wherever her tracks were the sugar was whitened. Experiments were instituted, and the result was that wet clay came to be used in refining sugar.

The art of etching upon glass was discovered by a Nuremburg glasscutter. By accident a few drops of aquafortis fell upon his spectacles. He noticed that the glass became corroded and softened where the acid had touched it. That was hint enough. He drew figures upon glass with varnish, applied the corroding fluid and then cut away the glass around the drawing. When the varnish was removed the figures appeared raised upon a dark ground.

The shop of a Dublin tobacconist, by the name of Lundyfoot, was destroyed by fire. While he was gazing dolefully into the smoldering ruins, he noticed that his poor neighbors were gathering the snuff from the canisters. He tested the snuff for himself, and discovered that the fire had largely improved its pungency and aroma. It was a hint worth profiting by. He secured another shop, built a quantity of ovens, subjected the snuff to a heating process, gave the brand a particular name, and in a few years became rich by an accident which at first he thought had completely ruined him.

The progress of languages spoken by different peoples is said to be as follows: English, which at the commencementthe of century was only spoken by 22,000,000, is now spoken by 90,000,000; Russian by 63,000,000, instead of 30,000,000; German by 66,000,000, instead of 38,000,000; Spanish by 44,000,000; instead of 32,000,000; Italian by 30,000,000, instead of 18,000,000; Portugese by 13,000,000, instead of 8,000,000. This is for England an increase of 3.10 per cent.; for Russia 1.10 per cent., for Germany, 70 per cent.; for Spain, 36 per cent., etc. In the case of France the increase has been from 34,000,000 to 46,000,000, or 36 per cent.

